# Supplementary material for: Organizational Learning in Healthcare Contexts after COVID-19: A Study of 10 Intensive Care Units in Central and Northern Italy through Framework Analysis
Source: Int J Environ Res Public Health. 2023 Sep 1;20(17):6699. doi: 10.3390/ijerph20176699 (PMC10487410; doi:10.3390/ijerph20176699)
Supplement: Supplementary file 1 [file ijerph-20-06699-s001.zip › ijerph-2480362-supplementary.pdf]

### Demographic and occupational data of the participant

## Interviews

| | | |
|---|---|---|
| Interviewee 1 | San Valentino Civil Hospital, Montebelluna | Head of ICU |
| Interviewee 2 | Merate Hospital | ICU nursing manager |
| Interviewee 3 | Maggiore Hospital, Bologna | ICU manager |
| Interviewee 4 | San Giovanni Bosco Hospital, Turin | ICU nursing manager |
| Interviewee 5 | Lecco Hospital | ICU nursing manager |
| Interviewee 6 | Azienda Ospedaliera S. Anna, Como | ICU nursing manager |
| Interviewee 7 | San Donato Hospital, Arezzo | ICU manager |
| Interviewee 8 | Maurizio Bufalini Hospital, Cesena | ICU nurse manager |
| Interviewee 9 | Maggiore della Carità Hospital, Novara | ICU physician in charge |
| Interviewee 10 | Misericordia Hospital, Grosseto | ICU manager |

## Focus groups

| | | |
|---|---|---|
| Focus group n.1 Maggiore Hospital, Bologna | Participant 1 | Psychologist-psychotherapist |
| | Participant 2 | Infectivologist |
| | Participant 3 | Physician |
| | Participant 4 | Physiotherapist |
| | Participant 5 | Physician |
| | Participant 6 | Healthcare assistant |
| | Participant 7 | Healthcare assistant |
| | Participant 8 | Physician |
| | Participant 9 | Physician |
| | Participant 10 | Nurse |
| | Participant 11 | Nurse |
| Focus group n.2 Misericordia Hospital, Grosseto | Participant 1 | Physician |
| | Participant 2 | Physician |
| | Participant 3 | Physician |
| | Participant 4 | Physician |
| | Participant 5 | ICU nursing manager |
| | Participant 6 | ICU nursing manager |
| | Participant 7 | Nurse |
| | Participant 8 | Trainee physician |
| | Participant 9 | Nurse |
| | Participant 10 | Healthcare assistant |
| | Participant 11 | Infectivologist |
| | Participant 12 | ICU manager |
| Focus group n.3 | Participant 1 | Physician |

| | | |
|---|---|---|
| Maggiore della Carità Hospital, Novara | Participant 2 | Physician |
| | Participant 3 | Physician |
| | Participant 4 | Trainee physician |
| | Participant 5 | ICU nursing manager |
| | Participant 6 | ICU nursing manager |
| | Participant 7 | Nurse |
| | Participant 8 | Nurse |
| | Participant 9 | Nurse |
| | Participant 10 | Nurse |
| | Participant 11 | Infectivologist |
| Focus group n.4 Lecco Hospital | Participant 1 | Nurse |
| | Participant 2 | Nurse |
| | Participant 3 | Healthcare assistant |
| | Participant 4 | Healthcare assistant |
| | Participant 5 | Infectivologist |
| | Participant 6 | Healthcare assistant |
| | Participant 7 | ICU nursing manager |
| | Participant 8 | ICU manager |
| | Participant 9 | Nurse |
| | Participant 10 | Psychologist-psychotherapist |
| | Participant 11 | Physician |
| | Participant 12 | Physician |
| | Participant 13 | Physician |
| Focus group n.5 Maurizio Bufalini Hospital, Cesena | Participant 1 | Physician |
| | Participant 2 | Physician |
| | Participant 3 | Nurse |
| | Participant 4 | Healthcare assistant |
| | Participant 5 | Physiotherapist |
| | Participant 6 | ICU nursing manager |
| | Participant 7 | ICU manager |
| | Participant 8 | Physician |
| | Participant 9 | Physician |
| | Participant 10 | Physician |
| | Participant 11 | Nurse |
| Focus group n.6 San Donato Hospital, Arezzo | Participant 1 | Head of ICU |
| | Participant 2 | Psychologist-psychotherapist |
| | Participant 3 | Physician |
| | Participant 4 | Physician |
| | Participant 5 | Physician |
| | Participant 6 | Nurse |
| | Participant 7 | ICU nursing manager |
| | Participant 8 | Nurse |
| | Participant 9 | Nurse |
| | Participant 10 | Healthcare assistant |
| | Participant 11 | Healthcare assistant |
| | Participant 12 | Infectivologist |
| Focus group n.7 | Participant 1 | ICU nursing manager |

| | | |
|---|---|---|
| San Valentino Civil Hospital, Montebelluna | Participant 2 | Nurse |
| | Participant 3 | Healthcare assistant |
| | Participant 4 | Nurse |
| | Participant 5 | Physiotherapist |
| | Participant 6 | Physician |
| | Participant 7 | Physician |
| | Participant 8 | Physician |
| Focus group n.8<br>San Giovanni Bosco Hospital, Turin | Participant 1 | Physician |
| | Participant 2 | Physician |
| | Participant 3 | Physiotherapist |
| | Participant 4 | Nurse |
| | Participant 5 | Nurse |
| | Participant 6 | ICU nursing manager |
| | Participant 7 | Physician |
| | Participant 8 | Healthcare assistant |
| | Participant 9 | Infectivologist |
| | Participant 10 | Healthcare assistant |
| Focus group n.9<br>Azienda Ospedaliera S. Anna, Como | Participant 1 | ICU nursing manager |
| | Participant 2 | Healthcare assistant |
| | Participant 3 | Infectivologist |
| | Participant 4 | Healthcare assistant |
| | Participant 5 | Physician |
| | Participant 6 | Physician |
| | Participant 7 | Nurse |
| | Participant 8 | Nurse |
| Focus group n.10<br>Merate Hospital (Lecco) | Participant 1 | Nurse |
| | Participant 2 | Nurse |
| | Participant 3 | Infectivologist |
| | Participant 4 | ICU nursing manager |
| | Participant 5 | Physician |
| | Participant 6 | Physician |
| | Participant 7 | Physician |
